# Hydrogen Promotes the Growth of Platinum Pyramidal Nanocrystals by Size-Dependent Symmetry Breaking

**DOI:** 10.1021/acs.nanolett.2c04982

**Published:** 2023-03-30

**Authors:** Diana Nelli, Valentina Mastronardi, Rosaria Brescia, Pier Paolo Pompa, Mauro Moglianetti, Riccardo Ferrando

**Affiliations:** ‡ Dipartimento di Fisica, Università di Genova, Via Dodecaneso 33, Genova 16146, Italia; § Istituto Italiano di Tecnologia, Nanobiointeractions & Nanodiagnostics, PVia Morego 30, Genova 16163, Italy; ⊥ BeDimensional S.p.A., Via Lungotorrente Secca 30R, Genova 16163, Italy; || Electron Microscopy Facility, Istituto Italiano di Tecnologia, Via Morego 30, Genova 16163, Italy; # Center for Cultural Heritage Technology, Istituto Italiano di Tecnologia, via Torino 155, Venice 30172, Italy

**Keywords:** platinum, hydrogen, nanoparticles, growth, symmetry breaking

## Abstract

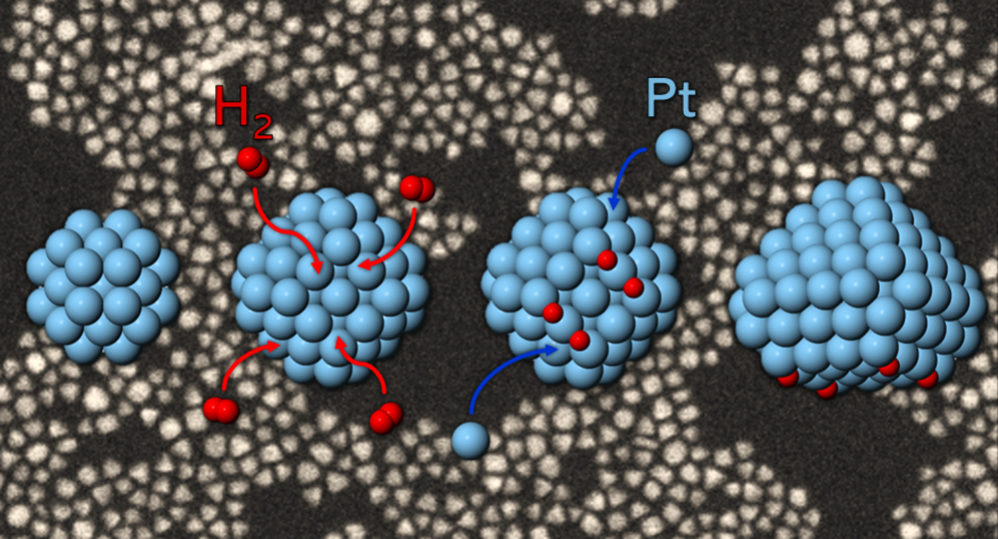

The growth of pyramidal platinum nanocrystals is studied by a combination of synthesis/characterization experiments and density functional theory calculations. It is shown that the growth of pyramidal shapes is due to a peculiar type of symmetry breaking, which is caused by the adsorption of hydrogen on the growing nanocrystals. Specifically, the growth of pyramidal shapes is attributed to the size-dependent adsorption energies of hydrogen atoms on {100} facets, whose growth is hindered only if they are sufficiently large. The crucial role of hydrogen adsorption is further confirmed by the absence of pyramidal nanocrystals in experiments where the reduction process does not involve hydrogen.

Polymer electrolyte membrane fuel cells (PEMFCs) have demonstrated superior advantages as power sources for the industrial sector with great potential in the heavy-duty and naval industries.^1−4^ However, major technological breakthroughs are needed for PEMFCs to become a viable alternative. A key issue is the tailored design of the nanocatalysts in the electrodes. Although the oxygen reduction reaction (ORR) can be catalyzed by non-Pt catalysts in alkaline conditions,^5^ superior performances are achieved in acidic proton-exchange systems where nanostructured platinum is necessary to overcome the sluggish kinetics of the ORR at the cathode.^1,6,7^ Due to the cost of Pt, there is an urgent need to maximize the utilization and longevity of the catalysts in PEMFC stacks.^8^ This is usually achieved by using small (3–5 nm) Pt nanoparticles supported on carbon black, commonly formed via processes resulting in polycrystalline Pt structures. However, these catalysts suffer from the degradation via corrosion of the carbon support or aggregation, migration, or dissolution of the Pt nanoparticles.^9,10^ Therefore, the large-scale synthesis of platinum nanocrystals with defined surface facets and, hence, with high mass activity and high stability is a particularly timely challenge.^11^

Another major challenge in the synthesis of surface-tailored catalytic nanoparticles is due to a geometrical issue:^12^ as the particle size decreases, the proportion of specific desired facets (e.g., {111} and {100}) tends to decrease dramatically due to the predominance of edges, steps, corners, and kinks, i.e., low coordination sites.^11^ Therefore, it is necessary to achieve high monodispersity through a careful design of the synthetic process in order to achieve the maximum possible flat surfaces. This is only possible by working on the complex dynamics of the synthesis process. Consequently, the design of Pt catalysts with sizes of a few nanometers and a high percentage of {111} facets is a complex challenge,^12^ which would be much better addressed with in-depth understanding of the key growth processes at the atomic/molecular level.

Very recently, a new one-pot aqueous phase process has allowed the production of well-controlled Pt pyramidal nanocrystals (nanopyramids in the following) with excellent catalytic properties and outstanding durability under repeated cycles.^6^ The nanopyramids are deemed ideal for application in PEMFCs because they present a high fraction of very well-defined {111} facets and a size distribution close to 3 nm, at which the mass activity of Pt for the ORR is known to be maximal. The experimental conditions for nanopyramid growth have been identified, but the relevant atomic-level mechanisms are still unclear. In fact, a nanopyramid is a peculiar type of truncated octahedron, where five tips out of six are fully grown to their completion, while the sole remaining tip has not grown. In principle, the six tips of the octahedron are all equivalent, so that this type of growth requires a nontrivial symmetry breaking. Such symmetry breaking was not observed in the growth of pure Pt nanocrystals in the gas phase,^13^ where either complete octahedra or tetrahedra were grown. Therefore, it must originate from the interaction between the Pt nanocrystals and their liquid-phase environment. In this work, we show by a combination of experiments and density functional theory (DFT) calculations that the role of hydrogen is crucial to the formation of nanopyramids. In addition, the DFT calculations indicate that the symmetry breaking is due to the size-dependent adsorption of hydrogen on the {100} facets of Pt.

Pyramidal Pt nanoparticles with a high fraction of {111} facets and size below 4 nm are obtained in water without the need for polymers, surfactants, and other sticky capping agents (see Figure 1a–c and Figure S1). Only two chemicals with synergistic functions are employed — sodium citrate and a strong reducing agent, sodium borohydride.^6^ The process achieves the fast and scalable production of the nanomaterial, in an airtight capped glass flask. The nanocrystals have pyramidal shape and narrow polydispersity as evident from high-resolution transmission electron microscopy (HR-TEM) (Figure 1a) and high-angle annular dark-field scanning TEM (HAADF-STEM) imaging (Figure 1c). The shape and faceting deduced from HR-TEM images is drawn in Figure 1b, showing four large {111} facets and five small {100} facets truncating the corners of the particle, with only one more extended {100} facet forming the base of the pyramid. The shape is shown in different views in Figure S2. The size distribution shows an average of 3.4 nm with a standard deviation of 0.4 nm (Figure 1d). Nanopyramids are predominant (more than 75%).

**Figure 1:**
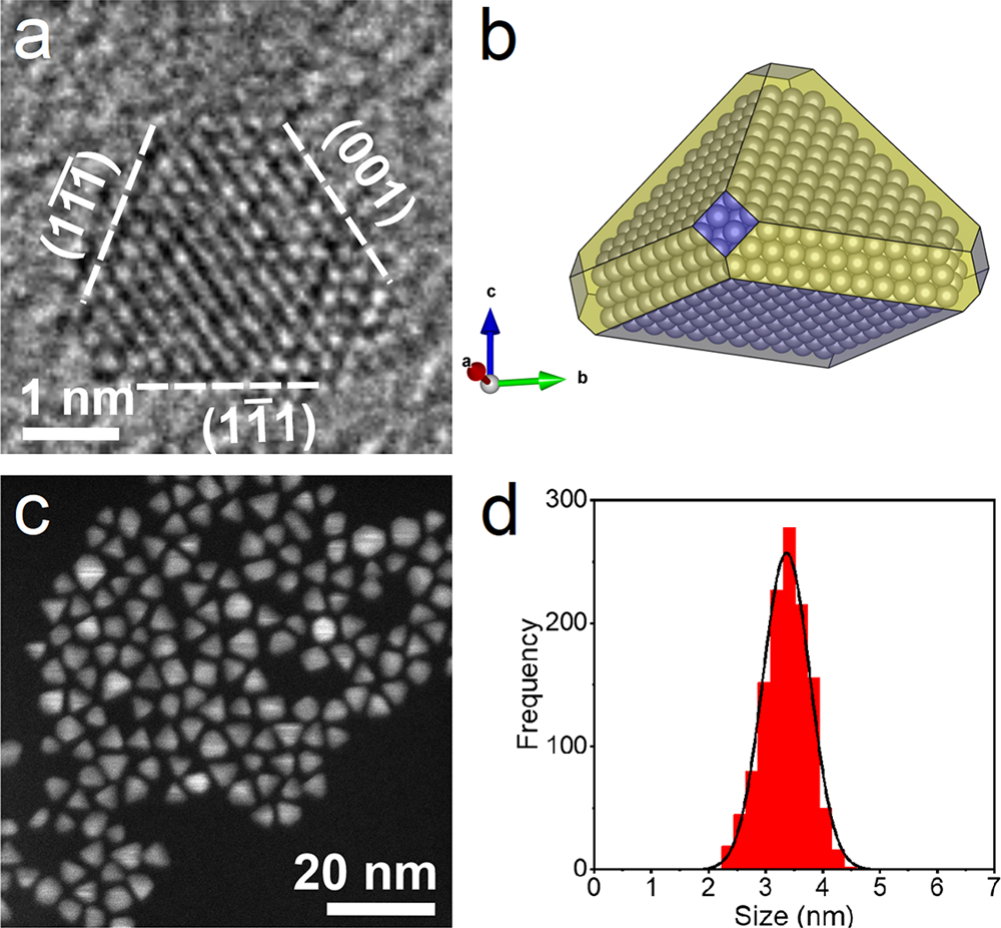
(a) HR-TEM image of a Pt pyramidal nanocrystal, with facets indexed according to fcc Pt (ICSD 41525), and (b) schematic drawing of the deduced shape and faceting, with the {111} facets in yellow and the {100} facets in blue, obtained by VESTA.^14^ (c) HAADF-STEM image of a group of pyramid nanocrystals and (d) the deduced size distribution.

Although only two chemicals are employed, their specific role and the reaction mechanism need to be elucidated. However, it is difficult to isolate the separate contributions experimentally as citrate molecules are also necessary to avoid nanoparticle aggregation and precipitation.

Previous results in the literature indicate that citrate favors the growth of Pt nanoparticles whose surface is mostly {111}, such as octahedra with small symmetric truncations and tetrahedra.^10,11,15^ We recall that growth in the gas phase leads to the same types of shape.^13^ Therefore, citrate is unlikely to cause the growth of the large {100} facets at the base of the nanopyramids. This hints at the possible key role of sodium borohydride, which dissociates in water according to NaBH_4_ + 2H_2_O → NaBO_2_ + 4H_2_, releasing hydrogen molecules in the solution.

The importance of hydrogen in shaping Pt nanocrystals was reported in ref (16), where it was demonstrated that, after the dissociation of the H_2_ molecule, hydrogen atoms preferentially adsorb on {100} faces of Pt. This has two important consequences on both equilibrium shapes and growth kinetics. First, for sufficiently high hydrogen pressure, hydrogen-covered {100} surfaces have a lower surface energy than hydrogen-covered {111} faces, so that the equilibrium shapes of the Pt nanocrystals shifts from the truncated octahedron^13^ to the cube, as follows from the equilibrium Wulff construction.^17^ Second, the preferential adsorption of hydrogen on {100} facets might hinder the accommodation of further incoming Pt atoms on these facets slowing down their growth. According to the kinetic Wulff construction,^17^ nongrowing facets expand at the expense of growing ones, leading to the shrinking of {111} facets and finally to the formation of cubic nanocrystals because all six {100} faces are equivalent. In summary, for both quasi-equilibrium and kinetically dominated growth, the final result for hydrogen-covered Pt nanocrystals is the cubic shape,^16^ which does not match with the formation of pyramidal nanocrystals.

A key point that is missing in both equilibrium and kinetic Wulff constructions is the full treatment of finite size effects. In small crystals such as our nanopyramids, these effects can be very important. Here we show that the finite size of the facets is crucial in determining the adsorption energy of hydrogen atoms, with clear consequences on the growth kinetics.

We study the adsorption of hydrogen atoms on the model Pt cluster shown in Figure 2. This cluster is a small nanopyramid, which contains {100} facets of sizes 2 × 2 and 3 × 3 atoms. The cluster has 75 atoms and diameter of 1.2 nm, being therefore smaller than the pyramidal clusters in the experiment. However, such a small cluster is representative of the early stages of the growth, at which we believe that the symmetry breaking starts.

**Figure 2:**
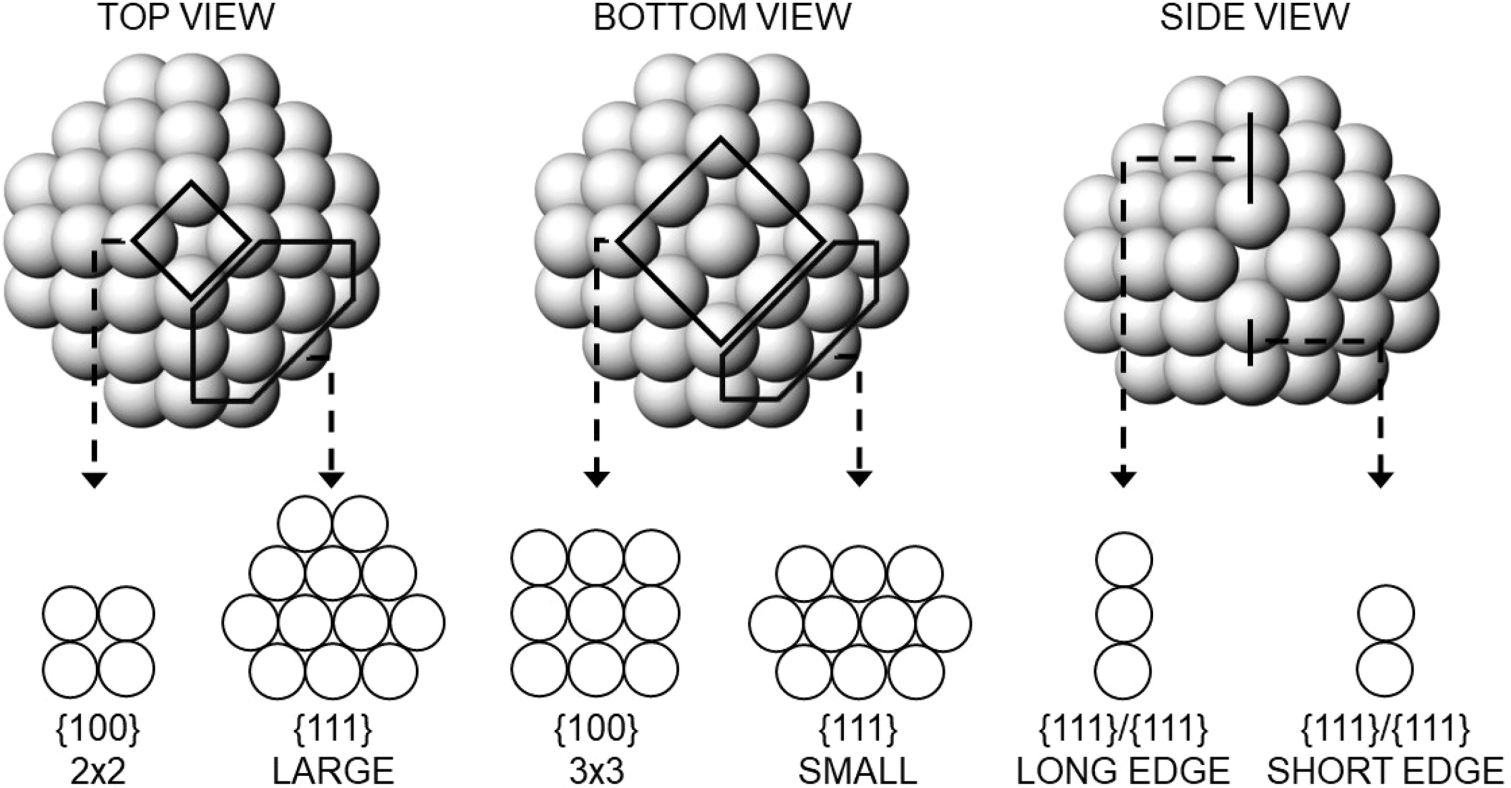
Pt cluster for DFT calculations. It contains 75 Pt atoms. In the top row three views of the cluster are reported, whereas the bottom row shows all its different facets.

As known from the literature,^18−20^ adsorption of H_2_ on Pt is dissociative with low dissociation barriers, allowing us to consider atomic hydrogen only in the following calculations. We calculate the adsorption energies of H on the cluster of Figure 2 by DFT (see the Methods section in the Supporting Information for details), making a systematic exploration of the possible adsorption sites for one, two and four hydrogen atoms. We restrict our calculations to surface sites since H absorption in inner sites was proved to be largely unfavorable compared to surface adsorption;^21,22^ therefore, inner sites begin to fill only when the cluster surface is saturated by H.^23−25^

The results for the adsorption of an isolated H atom are summarized in Table 1, with the corresponding configurations schematically shown in Figure 3. The best site for adsorption (site B in Figure 3) is a bridge site at the border of the 3 × 3 square facet, followed by bridge sites on the edges between {111} facets (sites 2 and 3 in Figure 3). Also on the 2 × 2 square facet at the top of the cluster, the most favorable site is of bridge type, but it is at the sixth place in the overall ranking. Adsorption on the 2 × 2 facet in on-top position (not shown in the figure) is not even stable, because the H atom shifts spontaneously from that position to the edge between nearby {111} facets (i.e., to position 8 in Figure 3).

**Figure 3:**
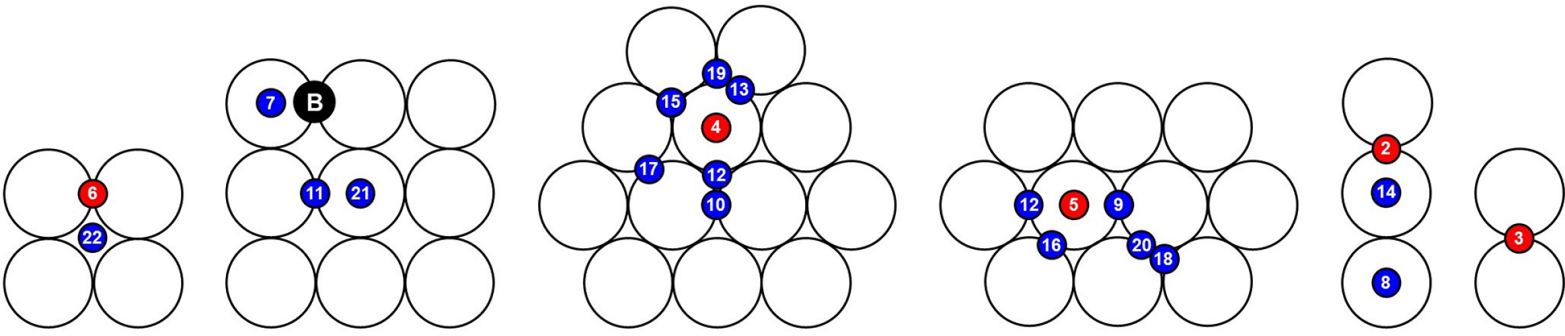
Adsorption sites for an isolated H atom. The numbering of the adsorption sites reflects their energetic ranking with respect to site B, which is the lowest-energy adsorption site for an isolated H atom (see Table 1).

**Table 1:** Energy of the Pt_75_ Cluster of Figure 2 with an Isolated H Atom Adsorbed on Its Surface In the Sites of Figure 3^a^

Ranking	Facet	Site	Δ*E* (eV)
BEST	{100} 3 × 3	br	0.000
2	{111}/{111} LE	br	0.046
3	{111}/{111} SE	br	0.060
4	{111} L	top	0.065
5	{111} S	top	0.065
6	{100} 2 × 2	br	0.084
7	{100} 3 × 3	top	0.118
8	{111}/{111} LE	top	0.123
9	{111} S	br	0.128
10	{111} L	br	0.175
11	{100} 3 × 3	br	0.179
12	{111} L	3-fold	0.200
13	{111} L	br	0.206
14	{111}/{111} LE	top	0.212
15	{111} L	3-fold	0.225
16	{111} S	br	0.242
17	{111} L	br	0.263
18	{111} S	3-fold	0.295
19	{111} L	3-fold	0.300
20	{111} S	br	0.311
21	{100} 3 × 3	top	0.360
22	{100} 2 × 2	4-fold	0.425

a The facets and edges are those of Figure 2, namely, {100} 2 × 2, {100} 3 × 3, {111} LARGE (L), {111} SMALL (S), {111}/{111} LONG EDGE (LE), and {111}/{111} SHORT EDGE (SE). Site types br, top, 3-fold, and 4-fold refer to bridge, on-top, three-fold, and four-fold sites, respectively. Δ*E* is the energy difference with respect to adsorption in the best site (site B in Figure 3).

The better energy for bridge adsorption on the 3 × 3 facet compared to the bridge on the 2 × 2 facet may be due to the different relaxation of Pt atoms on the two facets. In fact, on the 3 × 3 facet the distance between the two Pt atoms is slightly smaller than on the 2 × 2 facet, so that the H atoms can bind with slightly smaller bond lengths.

The preference for bridge adsorption of H on Pt clusters in this size range was demonstrated in experiments.^26^ Besides the adsorption on bridge sites, H atoms on on-top and 3-fold hollow sites were identified, while there was no evidence for adsorption in the 4-fold hollows.^26^ These experimental results very well agree with our data of Table 1. Previous DFT calculations for H on an octahedral Pt_85_ cluster,^27^ whose surface is made of {111} facets only, showed that the most favorable sites are bridges on the edges, followed by on-top and 3-fold hollow sites in the terraces. These data very well agree with our calculations.

In summary, our DFT results show that the most favorable adsorption sites for isolated H atoms are at the border of the 3 × 3 facet. Moreover, if we compare the best site for hydrogen adsorption of each facet/edge (i.e., the first six sites in Table 1), the site on the 2 × 2 facet turns out to be the least energetically favorable.

Here below we consider the adsorption of two and four H atoms on 2 × 2 and 3 × 3 facets. The main results are given in Figures 4 and 5. Complete data are reported in the Supporting Information.

**Figure 4:**
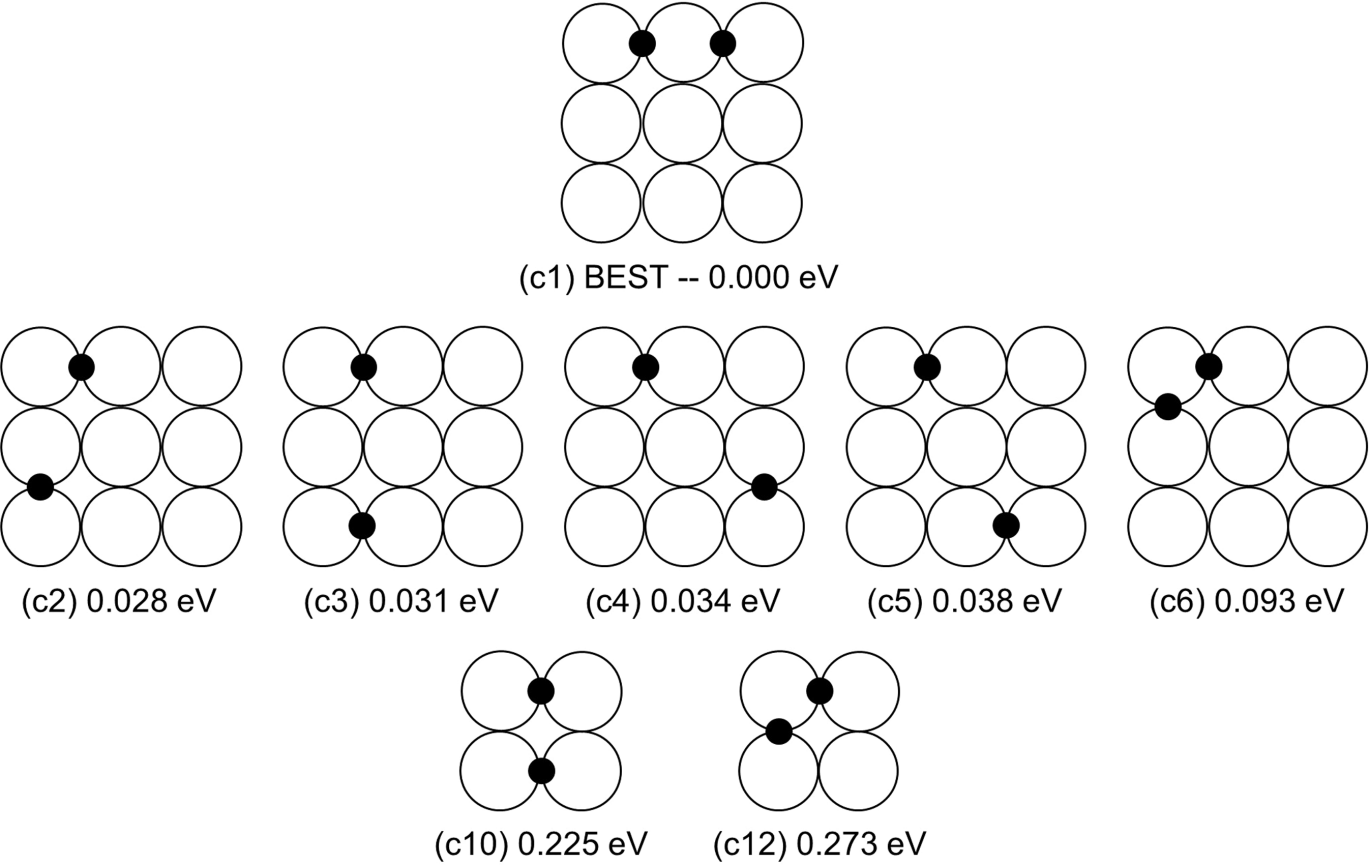
Adsorption sites for two H atoms in bridge configurations on the {100} facets. The configurations are ordered with increasing energy from the best one, whose energy is set to zero. For the 3 × 3 facet we show only the configurations within 0.1 eV from the best one. The complete data are reported in Figure S2.

**Figure 5:**
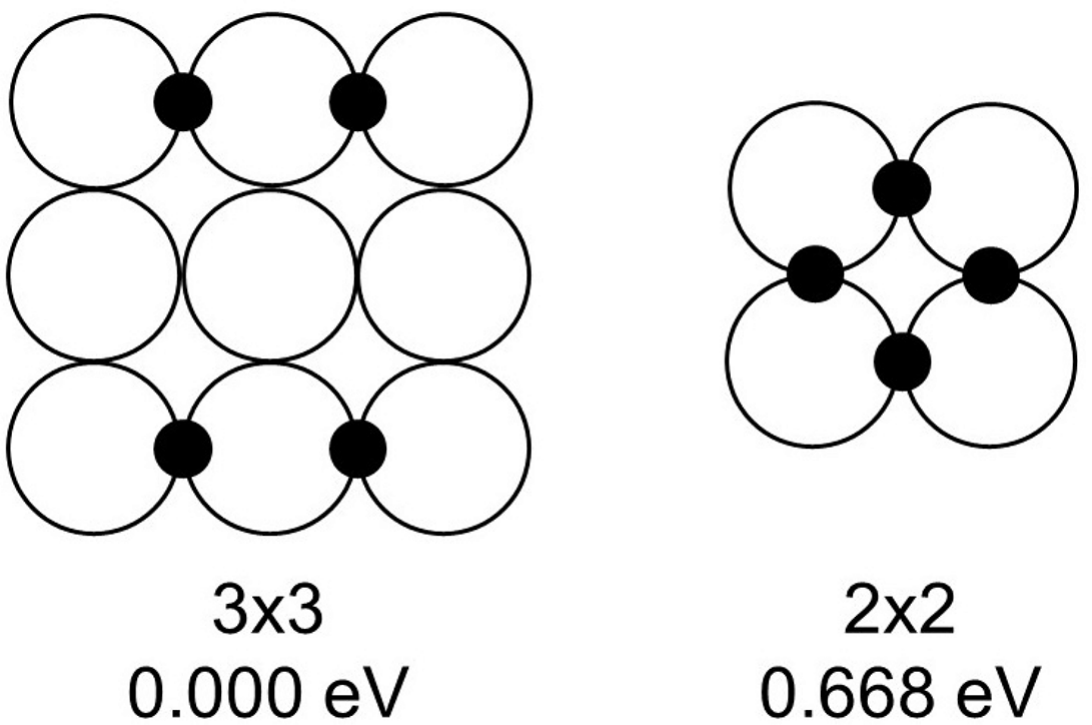
Adsorption sites for four H atoms in bridge configurations on the {100} facets.

The adsorption of two H atoms is especially important because it is likely that H_2_ molecules arrive on the surface of the Pt cluster and dissociate, therefore causing the adsorption of two H atoms at the same time. Here we consider the configurations in which both H atoms occupy bridge sites, since they are the most favorable for the adsorption. On the 2 × 2 facet, only two stable configurations are possible, while on the 3 × 3 facet, there are several different possibilities, the most important being shown in Figure 4. More complete data are reported in Figure S2.

In the best configuration (Figure 4c1), the two H atoms are placed in adjacent bridge positions along the same edge of the 3 × 3 facet. Configurations whose energy lies within 0.1 eV from the best one are shown in Figure 4c2–c6. Also in these cases, H atoms occupy bridges on the edges of the 3 × 3 facet. Configurations on the 2 × 2 facet are largely less favorable: the better of the two (Figure 4c10) is in tenth position in the overall ranking, 0.225 eV above c1. In addition, we have considered the adsorption of two H atoms on the large and small {111} facets and on the long {111}/{111} edge. These configurations turn out to be unfavorable compared to many of those on the 3 × 3 facet (see Figure S4), the best one being 0.112 eV above c1.

Let us estimate the occupation probability *p*_c1_ of the best configuration c1 with respect to the probability *p*_c10_ of the most favorable configuration on the 2 × 2 facet (c10). We assume that only one H_2_ molecule has dissociated on the surface, and that the atoms after dissociation are able to equilibrate on the cluster surface. Moreover, we assume that the vibrational partition function is the same for both configurations. This gives
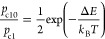
1where the factor 1/2 accounts for the different multiplicities of the configurations and Δ*E* = 0.225 eV. This expression gives *p*_c10_/*p*_c1_ = 8.3 × 10^–5^ and 7.3 × 10^–4^ at *T* = 300 and 400 K, respectively. These values indicate that atoms of the first H_2_ molecules that dissociate on the surface of the cluster are unlikely to accommodate on the small 2 × 2 facet, by far preferring the 3 × 3 facet. Also the probability of adsorption of two H atoms on the best sites of the {111} facets is low, 25 and 70 times smaller than *p*_c1_ at 400 and 300 K, respectively.

These considerations are reinforced by the data for the adsorption of four H atoms (see Figure 5), for which the energy difference Δ*E* between the best configurations on the two {100} facets is 0.668 eV.

Here we briefly discuss the dependence of our findings on nanoparticle size. We consider a Pt pyramid of 127 atoms presenting one larger {100} facet of size 4 × 4 (see Figure S5), which may form in a subsequent stage of the growth. Our DFT results (see the Supporting Information) show that adsorption of one, two and four H atoms on the 4 × 4 facet is even more favorable than on the 3 × 3, thus confirming the preference for larger {100} facets in the adsorption of hydrogen on top of Pt nanocrystals.

After unravelling the adsorption behavior of hydrogen on nanosized Pt, we investigate the effect of adsorbed hydrogen on the nanoparticle growth. It was suggested that hydrogen may hinder the adsorption of metal atoms, therefore affecting the growth kinetics. However, to the best of our knowledge, there are no clear results in the literature on this point. Here we study by DFT the adsorption of a Pt atom on the Pt cluster of Figure 2, considering both bare and hydrogen-covered configurations, the latter with two or four H atoms adsorbed on the 3 × 3 facet. We evaluate different Pt adsorption sites on the {111} and on the {100} facets, as shown in Figure S7. Complete data are reported and discussed in the Supporting Information. In Table 2 we compare the best sites for Pt adsorption on the different facets, for the bare cluster and for the cluster with four H atoms adsorbed on the 3 × 3 facet, as in Figure 5. The presence of H atoms strongly affects the adsorption of Pt. The adsorption site on the hydrogen-covered 3 × 3 facet is much more unfavorable compared to the same site of the bare cluster, becoming in close competition with sites on the {111} bare facets. The best adsorption site on the small {111} facet is even slightly lower in energy, and therefore more favorable for the accommodation of a Pt atom. For this reason, we expect the growth rate of the bare {111} facets and of the large hydrogen-covered {100} facet to be similar.

**Table 2:** Energy of the Pt Cluster of Figure 2 with One Pt Atom Adsorbed on Its Surface on the Different Facets^a^

	Δ*E* (eV)	
Facet	Pt on Pt_75_	Pt on Pt_75_+4H
{100} 2 × 2	0.000	0.000
{100} 3 × 3	0.429	0.867
{111} L	0.925	0.915
{111} S	0.882	0.852

a The two sets of data refer to the Pt adsorption on the bare cluster and on the cluster with four H atoms already adsorbed on the 3 × 3 facet, as in Figure 5. Δ*E* is the energy difference with respect to adsorption in the best site, i.e., on the 2 × 2 {100} facet. For the {111} facets different inequivalent adsorption sites are present (see Figure S7); here we report Δ*E* for site 2 in the large (L) {111} facet and for site 2 in the small (S) {111} facet. Complete data are reported in the Supporting Information.

Our data on hydrogen adsorption on top of Pt clusters and on Pt adsorption on top of hydrogen-covered Pt clusters indicate the following mechanism for the formation of nanopyramids. In the very first stage of the growth, small Pt clusters will form, exposing both {111} and {100} facets. A typical example of a small cluster of this type is the truncated octahedron of 38 atoms, whose surface contains eight hexagonal {111} facets and six square {100} facets of size 2 × 2. In the absence of hydrogen, this truncated octahedron grows naturally toward a complete octahedron. Even though some hydrogen atoms might eventually adsorb on the cluster, they would preferentially move to {111} facets, therefore not hindering the completion of the octahedral tips. However, the subsequent growth on top of the octahedron is likely to form new {100} facets, due to the occurrence of fluctuations during the process and because the formation of truncations is energetically favorable. When a first sufficiently large {100} facet is formed (our calculations show that 3 × 3 is enough), hydrogen preferentially adsorbs there, hindering the accommodation of further Pt atoms. This {100} facet will then grow with a similar rate as the {111} bare facets, becoming even more favorable for H adsorption when growing to 4 × 4 or larger. At the end of the growth, pyramidal nanoparticles will be formed, with one large {100} facet and {111} facets of almost equal size, as observed in the experiment.

By a careful analysis of the synthesis procedure, we proved that the growth of pyramids needs the hydrogen released by the decomposition of sodium borohydride to achieve narrow size distribution in size and shape. To reinforce this experimental evidence, we replace sodium borohydride with other reducing agents: hydroxylamine and formic acid. These two chemicals are widely used as they can reach the reducing power necessary to reduce the ionic precursors in solution to zerovalent metal. On the other hand, the reduction mechanisms are completely different from sodium borohydride^28−30^ and do not involve the formation of molecular hydrogen. Therefore, they are ideal control chemicals for establishing the role of hydrogen in the Pt pyramids growth.

The reaction with hydroxilamine does not produce any anisotropy. Moreover, aggregation and agglomeration occur, suggesting that the reaction does not follow the same pathway as in the case of sodium borohydride (Figure S8a, b). Also formic acid promotes the growth of nanoparticles with high polydispersion in size and shape (Figure S8c, d). We can therefore conclude that formic acid and hydroxilamine are not able to promote the formation of nanopyramids due to different reduction mechanisms in place. These experimental data further support the key role of hydrogen in promoting the growth of Pt pyramids, showing that the anisotropic growth cannot be explained using previously proposed general theories, as different reducing agents do not replicate the results achieved with sodium borohydride.

In summary, both experiments and DFT calculations support the evidence in favor of the key role of hydrogen in causing the formation of pyramidal nanocrystals. DFT calculations give atomic-level insight on the peculiar symmetry breaking needed for the formation of pyramids, indicating that it is due to the size-dependent energetics of hydrogen adsorption on the {100} facets. The role of hydrogen is further confirmed by the absence of pyramidal growth when other reducing agents are used. These results give a striking evidence of the importance of finite-size effects in the formation of nanostructures, and as such, they can provide a useful guide for better control of growth and synthesis at the nanoscale.

## Supporting Information Available

Experimental methods, details on DFT calculations, additional BF-TEM and HAADF-STEM images of pyramidal nanocrystals, 3D model of the pyramidal shape, complete DFT data for the adsorption of hydrogen atoms on Pt_75_ and on Pt_127_, complete DFT data for the effect of hydrogen on the adsorption of platinum, and experimental images of the Pt nanoparticle synthesis with hydroxilammine or formic acid (PDF)
