# Supplementary material for: Hydrogen Promotes the Growth of Platinum Pyramidal Nanocrystals by Size-Dependent Symmetry Breaking
Source: Nano Lett. 2023 Mar 30;23(7):2644–50. doi: 10.1021/acs.nanolett.2c04982 (PMC10103309; doi:10.1021/acs.nanolett.2c04982)
Supplement: Supplementary file 1 — nl2c04982_si_001.pdf [file nl2c04982_si_001.pdf]

Supporting Information for Hydrogen Promotes the Growth of Platinum Pyramidal Nanocrystals by Size-Dependent Symmetry Breaking

Diana Nelli,[†,⊥] Valentina Mastronardi,[‡,¶,⊥] Rosaria Brescia,[§] Pier Paolo Pompa,[‡] Mauro Moglianetti,[*,‡,∥] and Riccardo Ferrando[*,†]

†*Dipartimento di Fisica, Università di Genova, Via Dodecaneso 33, 16146 Genova, Italia*

‡*Istituto Italiano di Tecnologia, Nanobiointeractions&Nanodiagnostics, Via Morego 30 – 16163 Genova, Italy*

¶*BeDimensional S.p.A., Via Lungotorrente Secca 30R, 16163 Genova, Italy*

§*Electron Microscopy Facility, Istituto Italiano di Tecnologia, Via Morego 30, 16163 Genova, Italy*

∥*Center for Cultural Heritage Technology, Istituto Italiano di Tecnologia, via Torino 155, 30172 Venice, Italy.*

⊥*equally contributing*

E-mail: mauro.moglianetti@iit.it; ferrando@fisica.unige.it

Methods

Experimental

The 3 nm pyramidal Pt nanoparticles were synthesized using the method described in Mastronardi et al.[1] The synthesis results fast and easy, using only a small quantity of platinum

precursor and a 22 mM aqueous solution of $NaBH_4$ that is the donor of ions H+.

HR-TEM analysis was carried out on an image-Cs-corrected JEOL JEM-2200FS TEM, operated at 200 kV. BF-TEM and HAADF-STEM overview images were acquired on a Tecnai F20 TEM, operated at 200 kV. For TEM analyses, a small volume of the Pt nanoparticles suspension was drop-cast onto an ultrathin carbon/holey carbon-coated Cu grid.

DFT calculations

All DFT calculations were made by the open-source QUANTUM ESPRESSO software[2] using the Perdew-Burke-Ernzerhof exchange-correlation functional.[3] The convergence thresholds for the total energy, total force, and for electronic calculations were set to 10^{-4} Ry, 10^{-3} Ry/a.u. and 5×10^{-6} Ry respectively. We used a periodic cubic cell, whose size was set to 30 Å. Cutoffs for wavefunction and charge density were set to 46 and 401 Ry, according to Pt.pbe-n-kjpaw_psl.1.0.0.UPF and H.pbe-kjpaw_psl.0.1.UPF, as provided by the QUANTUM ESPRESSO pseudopotential library available at
`http://pseudopotentials.quantum-espresso.org/legacy_tables/ps-library/pt` and
`http://pseudopotentials.quantum-espresso.org/legacy_tables/ps-library/h`.

In our calculations, we first relaxed the bare Pt cluster, then we placed the H atoms manually at distances of about 1.5 Å from the closest Pt atom, and finally the system was again relaxed.

BF-TEM and HAADF-STEM images of pyramidal nanocrystals

The analysis of the bright-field transmission electron microscopy (BF-TEM) (Figure S1 a, b) and of the high-angle annular dark-field scanning TEM (HAADF-STEM) (Figure S1 c, d) images clearly proves the pyramidal shape for the vast majority of the nanoparticles in the sample and the narrow polydispersity.

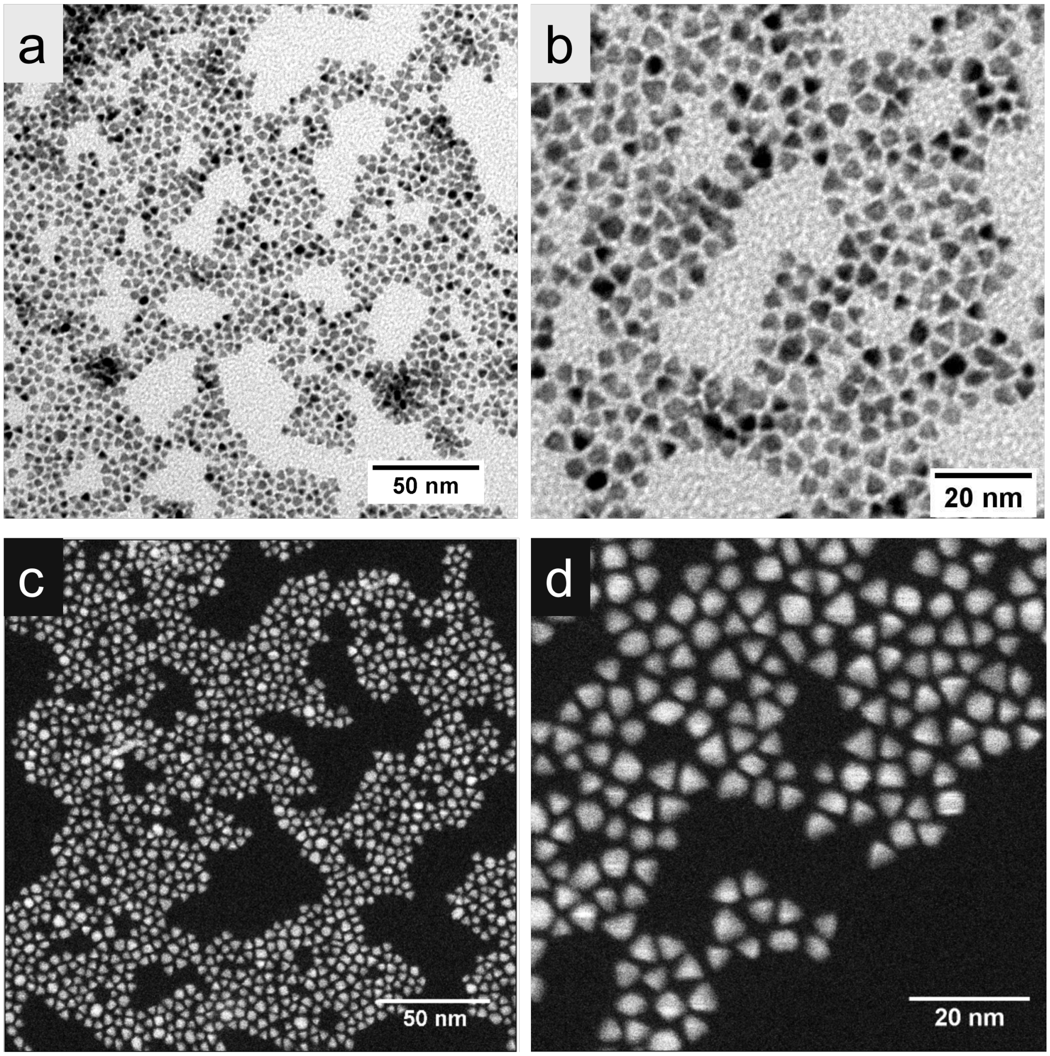

Figure S 1: (a, b) BF-TEM and (c, d) HAADF-STEM images of pyramidal nanocrystals at different magnification.

Shape and faceting of nanocrystals deduced from HR-TEM images

In Figure S2 we show the pyramidal shape deduced from HR-TEM image of the nanocrystal in Figure 1a in the main text. The shape is shown in different views, and the different type of surface facets (i.e. {111} and {100}) are highlighted.

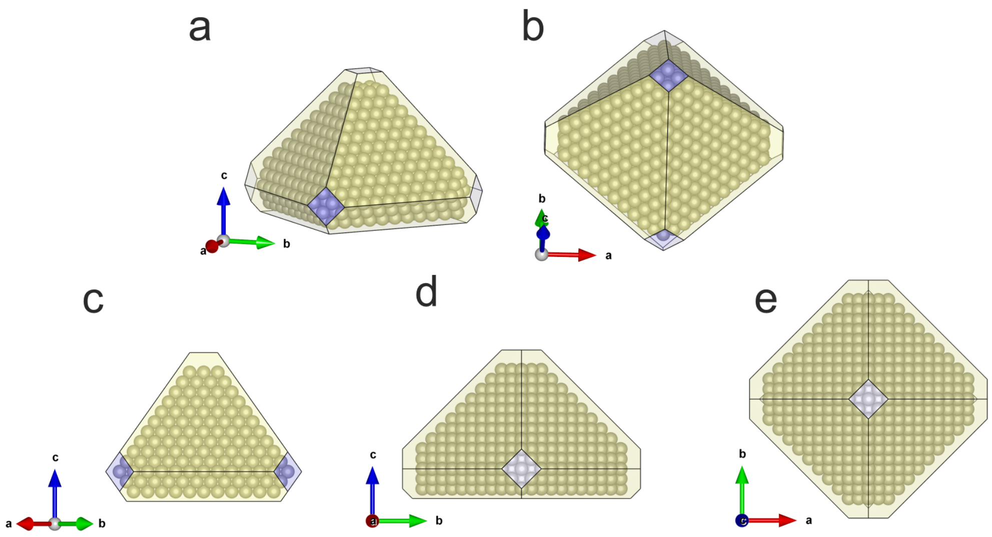

Figure S 2: Various views of the shape deduced for Pt pyramidal nanocrystals, as reported in Figure 1 in the main text, with the {111} facets in yellow and the {100} facets in blue, obtained by VESTA:[4] (a) view from the side, (b) from top, (c) along {110}, (d) along {100} and along {001}.

DFT data for the adsorption of two H atoms

In Figure S3 we schematically show all possible inequivalent adsorption configurations for two H atoms in bridge positions on 3×3 and 2×2 {100} facets. The configurations are ordered with increasing energy from the best one, whose energy is set to zero.

We studied the simultaneous adsorption of two H atoms also on the {111} facets and on the edge between two large {111} facets. In all cases, we considered the most favorable sites as found for the adsorption of a single H atom, i.e. on-top sites on the {111} facets

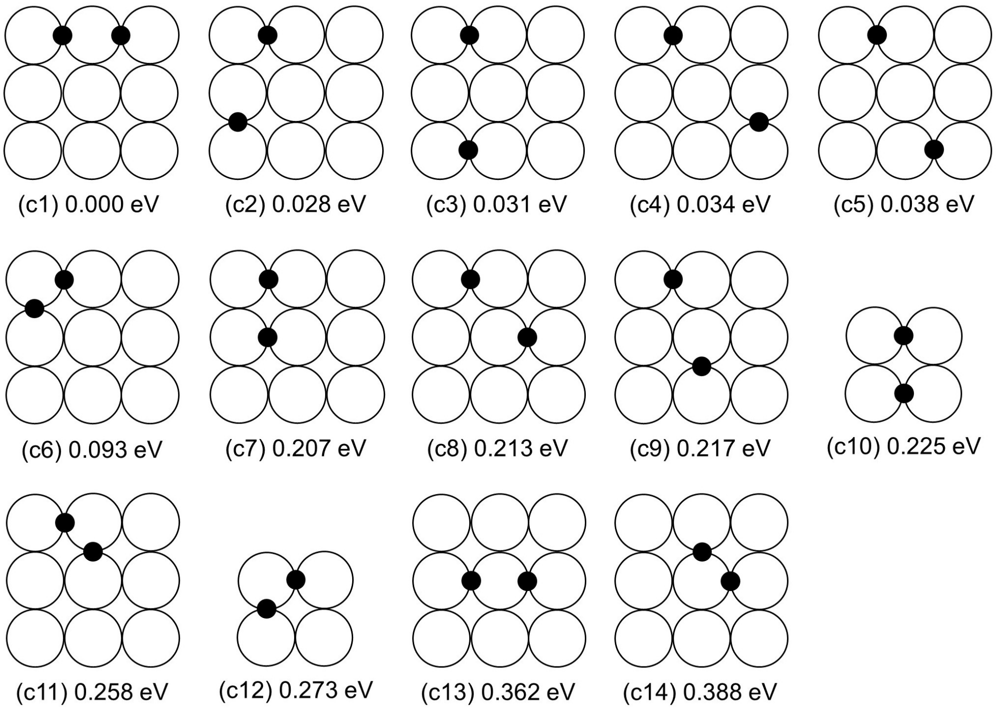

Figure S 3: Adsorption sites for two H atoms in bridge configurations on the {100} facets of size 3×3 and 2×2. The configurations are ordered with increasing energy from the best one, whose energy is set to zero.

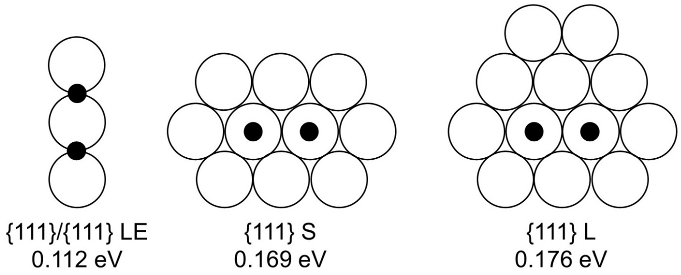

Figure S 4: Adsorption sites for two H atoms on the large (L) and small (S) {111} facet and on the long edge (LE) between two large {111} facets. The energy difference from the best configuration for the adsorption of two H atoms (configuration c1 in Figure S3) is reported below each configuration.

and bridge sites on the edge (see Figure 3 and Table 1 in the main text). The considered configurations and their corresponding energies are reported in Figure S4. Our results show that the {111} facets and the long edge are less favorable for the adsorption of two H atoms compared to the 3×3 {100} facet. Indeed, six configurations with H atoms adsorbed on the 3×3 facet (configurations from c1 to c6 in Figure S3) are lower in energy.

Hydrogen adsorption behavior on a Pt nanocrystal of larger size

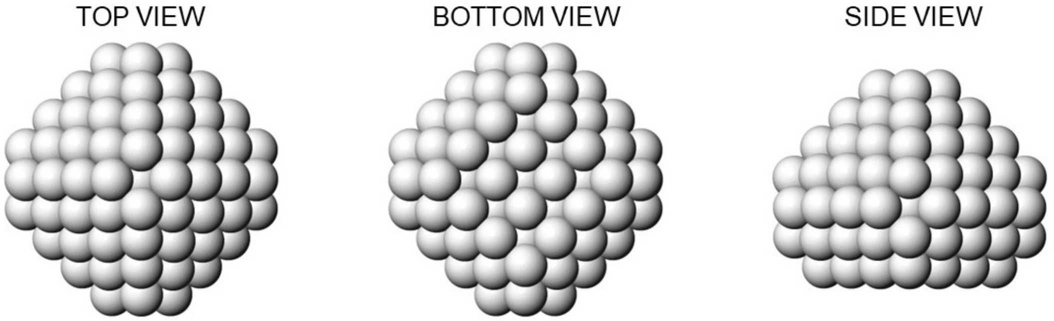

Figure S 5: Pt cluster of size 127 atoms employed for DFT calculations. The cluster is displayed in different views, showing the different types of surface facets. The base of the pyramid is a {100} facet of size 4×4, whereas all other {100} facets are of size 2×2.

We checked the validity of our results on a cluster of larger size, which is representative of a subsequent stage of the nanoparticle growth process. We considered a Pt pyramidal cluster of 127 atoms (corresponding to a diameter of 1.6 nm), with one large 4×4 {100} facet and five small 2×2 {100} facets. The cluster is shown in different views Figure S5. We evaluated different sites for the adsorption of one H atom and for the simultaneous adsorption of two and four H atoms on the 4×4 and on the 2×2 facets. We considered the most energetically favourable sites according to the results for the 75-atom cluster, i.e. bridge sites on the border of the facet. The energy of the different configurations were calculated by DFT, as explained in the Method section. The results for the adsorption of one, two and four H atoms are shown in Figure S6. For the adsorption of one H atom, we checked the two inequivalent

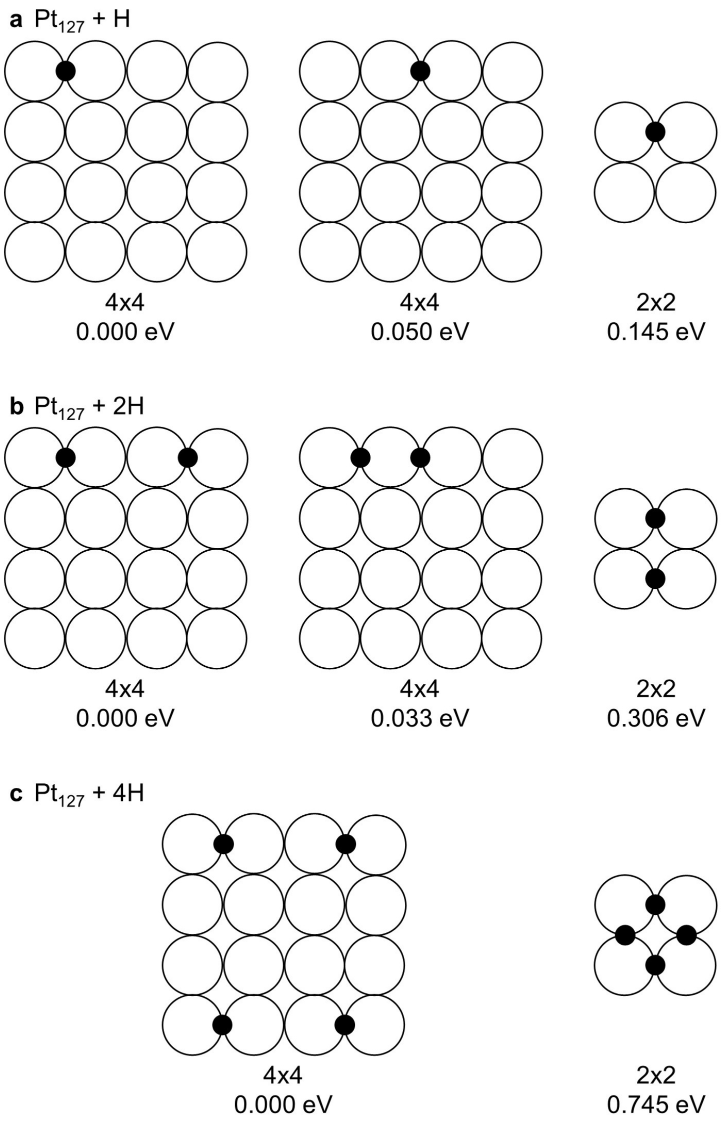

Figure S 6: Adsorption sites for (a) one H atom, (b) two H atoms and (c) four H atoms on the {100} facets of the 127-atom Pt cluster of Figure S5. In each case the configurations are oredered with increasing energy from the best one, whose energy is set to zero.

bridge sites on the edge of the 4×4 facet (see Figure 6a). For the adsorption of two H atoms, we checked the two inequivalent configurations with the H atoms adsorbed on bridge sites of the same edge of the 4×4 facet (see Figure 6b), which are expected to be the most favorable ones according to the results for H adsorption on the 3×3 facet (configuration c1 in Figure 4 of the main text). In both cases, configurations with H atoms adsorbed on the 4×4 facet are close in energy, and much more favorable than configurations with H adsorbed on the 2×2 facet. The same trend is found for the adsorption of four H atoms. We note that, in all cases, the energy difference between adsorption sites on the 2×2 and on the 4×4 facet is even larger than the one between 2×2 and 3×3 facets, as evaluated for the 75-atom cluster (see Table 1 and Figures 4 and 5 in the main text). Therefore, the overall picture arising from the calculations on the 75- and 127-atom clusters is that {100} facets of size 2×2 are by far the least favourable for hydrogen adsorption, followed by the 3×3 ones and then by the 4×4 ones. The comparison of the {100} facets of different sizes is summarized in Table S1.

Table S 1: Adsorption of H atoms on {100} facets of different sizes. For each number of H atoms adsorbed, we consider the most favourable configuration of each facet, and we compare their energy. The energy of the best configuration (which is always on the 4×4 facet) is set to zero. Data are taken from Table 1, Figure 4 and Figure 5 of the main text and from Figure S6.

Facet	Pt + H	Pt + 2H	Pt + 4H
	ΔE (eV)		
4×4	0.000	0.000	0.000
3×3	0.061	0.081	0.077
2×2	0.145	0.306	0.745

Effect of hydrogen on the adsorption of Pt atoms

We studied the effect of hydrogen on the adsorption of one Pt atom on the surface of the Pt cluster of size 75 atoms, shown in Figure 2 of the main text. Firstly, we considered the bare cluster, i.e. with no H atoms on its surface, and we evaluated all inequivalent adsorption

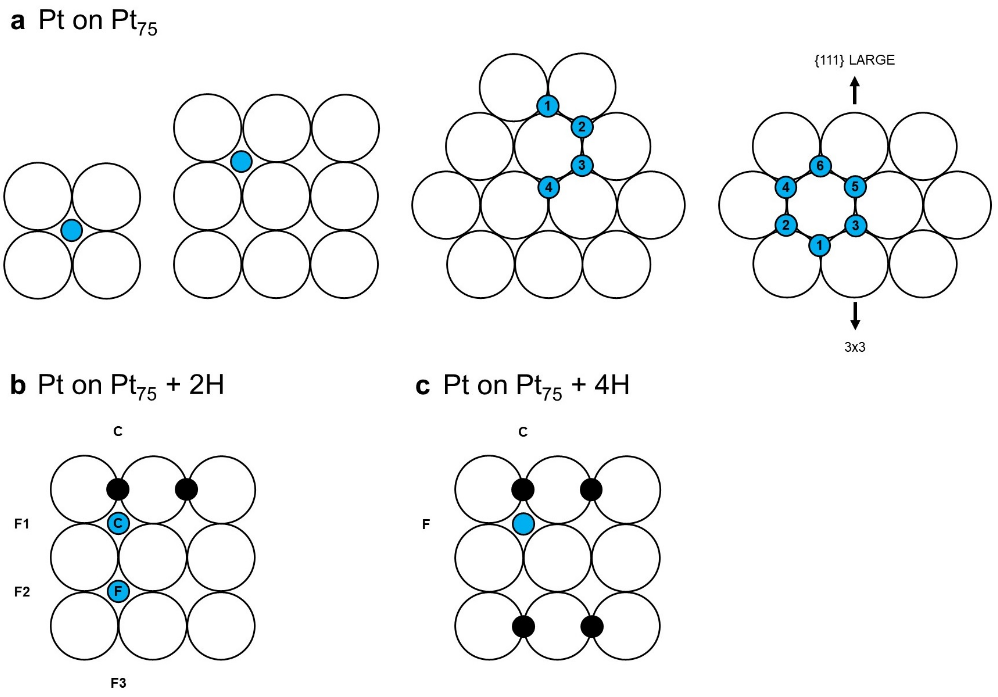

Figure S 7: Sites for the adsorption of one Pt atom on the Pt cluster of size 75 atoms. (a) Bare cluster; (b) cluster with two H atoms adsorbed on the 3×3 {100} facet, in the lowest-energy configuration (see Figure 4 of the main text); (c) cluster with four H atoms adsorbed on the 3×3 {100} facet. Sites for Pt adsorption are displayed as blue dots, whereas adsorbed H atoms are displayed as black dots. In (b): there are two sites for Pt adsorption on the 3×3 facet, namely close (C) or far (F) from the H atoms; sites 1 and 2 on the small {111} facet can be taken on different facets around the 3×3 one, with different distances from the H atoms (C, F1, F2 and F3). In (c): sites 1 and 2 on the small {111} facet can be taken on different facets around the 3×3 one, with different distances from the H atoms (C and F).

sites for a further Pt atom on the {100} and on the {111} facets, as shown on Figure S7 a. The energy of the different configurations was calculated by DFT, as explained in the Method section. Results are summarized in Table S2. The most favorable adsorption sites are those on the {100} facets. The best site is on the 2×2 facet. The site on the 3×3 facet is higher in energy, but much more favourable compared to all sites on the {111} facets, in agreement with the results in the literature.

We repeated the same calculations, but with two or four H atoms previously adsorbed on the {100} facet of size 3×3. In the case of two H atoms, we considered the lowest-energy configurations according to our previous calculations (see Figure 4 in the main text). In this case there are two inequivalent sites for Pt adsorption on the 3×3 facet, with different distances from the H atoms (see Figure S7 b). We evaluated two sites on the large {111} facet (sites 1 and 2 in Figure S7 a), and two sites on the small {111} facet (sites 1 and 2 in Figure S7 a). Sites on the small {111} facet are close to the border of the 3×3 facet, therefore different configurations are possible, in which the adsorbed Pt atom has different distances from the H atoms, as schematically shown in Figure S7 b. Results are summarized in Table S3. The presence of H atoms strongly affects the adsorption of Pt. For each given site type, sites closer to the H atoms are more energetically unfavorable. We especially note the two adsorption sites on the 3×3 facet have very different energy. The site far from the H atoms is equivalent to the corresponding site in the bare cluster, since we have calculated basically the same energy difference compared to the site on the 2×2 facet. On the other hand, the energy difference is almost doubled for the site close to the H atoms, which is therefore much more unfavorable, and in close competition with adsorption sites on the nearby {111} facets.

The same behavior is found when four H atoms are adsorbed on the 3×3 facet. Sites and corresponding energies are shown in Figure S7 c and in Table S4. In this case, one of the sites on the small {111} facet (site 2, far from the H atoms) is slightly more favorable than the site on the 3×3 facet. We recall that, in bare clusters, the adsorption of metal atoms is always more favorable on surface facets of {100} type compared to {111}, due to the higher

Table S 2: Energy of the Pt_{75} cluster of Figure 2 in the main text, with one Pt atom adsorbed on its surface, in the sites of Figure S7a. ΔE is the energy difference (in eV) with respect to adsorption in the best site, i.e. on the 2×2 {100} facet.

Pt on Pt_{75}		
Site		ΔE (eV)
{100} 2×2		0.000
{100} 3×3		0.429
{111} L	1	1.027
{111} L	2	0.925
{111} L	3	1.180
{111} L	4	1.283
{111} S	1	0.985
{111} S	2	0.882
{111} S	3	1.140
{111} S	4	1.045
{111} S	5	1.102
{111} S	6	0.812

Table S 3: Energy of the Pt_{75} cluster with two H atoms adsorbed on the 3×3 (see Figure 4(c1) in the main text), and with one further Pt atom adsorbed on its surface, in the sites of Figure S7b. ΔE is the energy difference (in eV) with respect to adsorption in the best site, i.e. on the 2×2 {100} facet.

Pt on Pt_{75}+2H		
Site		ΔE (eV)
{100} 2×2		0.000
{100} 3×3	F	0.427
{100} 3×3	C	0.858
{111} L	1	1.009
{111} L	2	0.926
{111} S	1 C	1.412
{111} S	1 F1	1.148
{111} S	1 F2	1.084
{111} S	1 F3	0.963
{111} S	2 C	0.938
{111} S	2 F1	0.872
{111} S	2 F2	0.864
{111} S	2 F3	0.856

Table S 4: Energy of the Pt_{75} cluster with four H atoms adsorbed on the 3×3 (see Figure 5 of the main text), and with one further Pt atom adsorbed on its surface, in the sites of Figure S7b. ΔE is the energy difference (in eV) with respect to adsorption in the best site, i.e. on the 2×2 {100} facet.

Pt on Pt_{75}+4H		
Site		ΔE (eV)
{100} 2×2		0.000
{100} 3×3		0.867
{111} L	1	1.003
{111} L	2	0.915
{111} S	1 C	1.376
{111} S	1 F	1.140
{111} S	2 C	0.917
{111} S	2 F	0.852

number of bonds that the adsorbed atom can form. Here we demonstrated that the presence of H atoms induce a reversal of this general trend.

Nanoparticle synthesis with hydroxilammine or formic acid

Pt pyramids were synthesized by adding 11 µL of H_2PtCl_6 (0.5 M) (Sigma-Aldrich) to 20 mL of MilliQ water at room temperature, immediately followed by a quick addition of 440 µL solution containing 0.03 M sodium citrate and 2 mM citric acid and 2 mL of either freshly prepared hydroxilammine (0.5 M) or formic acid (0.5 M). The vessel was placed in glycerol bath already at 90 °C and wait 10 minutes for the reaction under magnetic stirring at moderate rate. after this time the vessel was removed and left to cool under stirring for another hour.

BF-TEM images (Figure S8) clearly show that hydroxilammine and formic acid are not able to promote the growth of pyramidal nanocrystals.

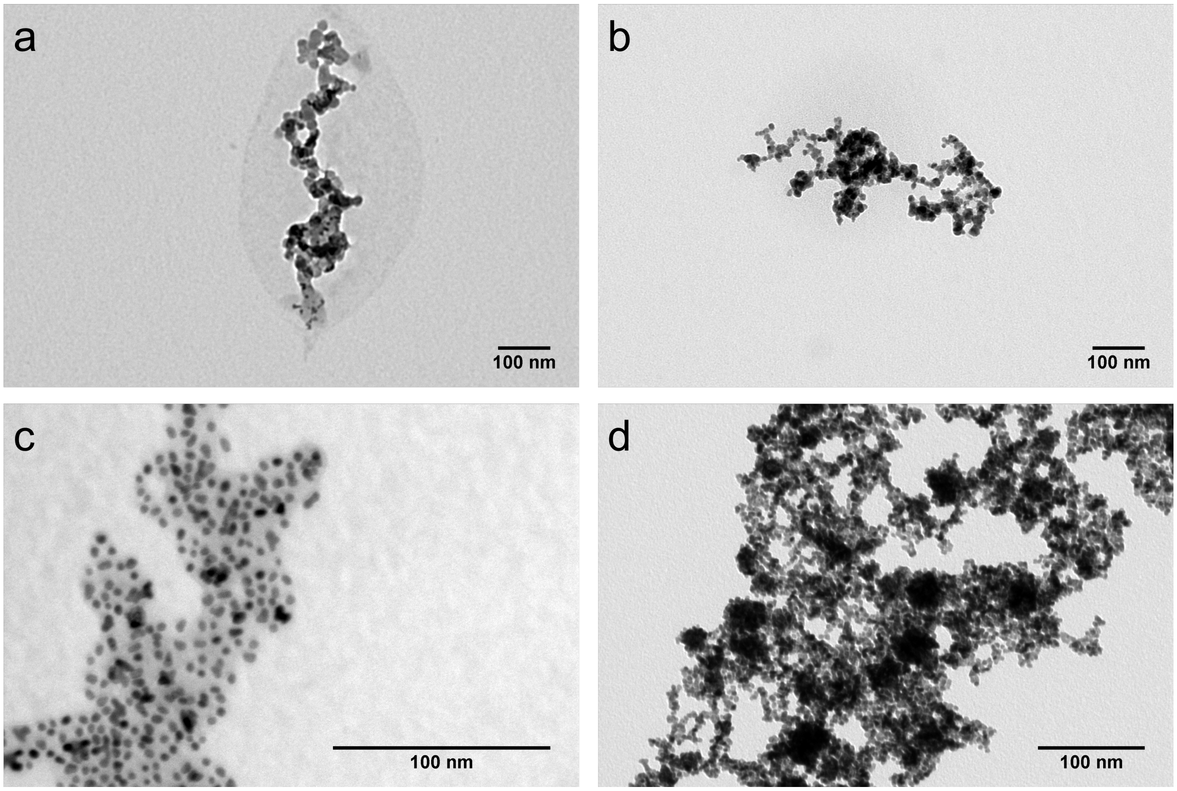

Figure S 8: BF-TEM images of and aggregate nanoparticles synthesized by using (a, b) hydroxilammine and (c, d) formic acid instead of sodium borohydrade.

References

(1) Mastronardi, V.; Magliocca, E.; Gullon, J. S.; Brescia, R.; Pompa, P. P.; Miller, T. S.; Moglianetti, M. Ultrasmall, Coating-Free, Pyramidal Platinum Nanoparticles for High Stability Fuel Cell Oxygen Reduction. *ACS Applied Materials & Interfaces* **2022**, *14*, 36570–36581.

(2) Giannozzi, P. et al. QUANTUM ESPRESSO: a modular and open-source software project for quantum simulations of materials. *J. Phys. Condens. Matter* **2009**, *21*, 395502.

(3) Perdew, J. P.; Burke, K.; Ernzerhof, M. Generalized gradient approximation made simple. *Phys. Rev. Lett.* **1996**, *77*, 3865–3868.

(4) Momma, K.; Izumi, F. *VESTA3* for three-dimensional visualization of crystal, volumetric and morphology data. *Journal of Applied Crystallography* **2011**, *44*, 1272–1276.
